# Trehalose Neuroprotective Effects on the Substantia Nigra Dopaminergic Cells by Activating Autophagy and Non-canonical Nrf2 Pathways

**DOI:** 10.22037/ijpr.2019.2387

**Published:** 2019

**Authors:** Shahram Darabi, Ali Noori-Zadeh, Hojjat-Allah Abbaszadeh, Farzad Rajaei, Salar Bakhtiyari

**Affiliations:** a *Cellular and Molecular Research Center, Qazvin University of Medical Sciences, Qazvin, Iran.*; b *Department of Clinical Biochemistry, Faculty of Allied Medical Sciences, Ilam University of Medical Sciences, Ilam, Iran. *; c *Hearing Disorders Research Center, Loghman Hakim Medical Center, Shahid Beheshti University of Medical Sciences, Tehran, Iran. *; d *Department of Anatomical Sciences and Biology, School of Medicine, Shahid Beheshti University of Medical Sciences, Tehran, Iran.*; e *Department of Clinical Biochemistry, Faculty of Medicine, Ilam University of Medical Sciences, Ilam, Iran.*

**Keywords:** Autophagy, Non-canonical Nrf2 pathway, p62, Parkinson′s disease, Trehalose

## Abstract

Trehalose, as a natural disaccharide, is known as an autophagy inducer. The neuroprotective effects of trehalose in the rat model of Parkinson′s disease were the aim of the present study. Parkinson′s disease model was induced by injecting 6-hydroxydopamine (6-OHDA) in the striatum of male Wistar rats. Apomorphine-induced behavior and substantia nigra neuronal counts were applied to evaluate the neuroprotective effects of trehalose. The autophagy was studied using the expression of p62 and LC3II/LC3I ratio. In addition, the antioxidant effects of trehalose were assessed by analyzing the levels of nuclear factor (erythroid-derived 2)-like 2 (Nrf2) and also glutathione reductase (GR), glutathione peroxidase (GPx) and Catalase (CAT) enzymes. Moreover, the levels of 3, 4-dihydroxyphenylacetic acid (DOPAC) and dopamine (DA) were assessed.The behavioral test showed that trehalose in the treatment group reduced the damage to the substantial nigra dopaminergic neurons, which was characterized by improved motor and reduced rotations in the treatment group as compared with the lesion group. In the histological examinations of the treatment group, trehalose prevented the destruction of dopaminergic neurons. Trehalose treatments increased autophagy (high LC3II/LC3I ratio) and the expression of the p62 protein as well. Through p62-dependent manner, it led to increased nuclear translocation of Nrf2 transcription factor and elevated expression of downstream antioxidant enzymes, such as GR, GPx, and CAT, restoring DA and DOPAC contents of the cells. In the current study, trehalose simultaneously protects substantia nigra dopaminergic cells by activating both non-canonical p62/SQSTM1-Keap1-Nrf2 and autophagy pathways.

## Introduction

Trehalose is a disaccharide containing two molecules of D-glucose. As a chemical chaperone, it protects the cells by stabilization of the cell membranes and macromolecules, such as proteins under oxidative stress conditions (1, 2). Moreover, trehalose possesses indirect inductive roles in autophagy through mTOR pathway (3). Studies have demonstrated that trehalose impedes amyloid accumulation and poly-glutamine extension of Huntington protein (4, 5). In fact, trehalose by inducing autophagy removes aggregated proteins in the neurodegenerative diseases for example, it inhibits alpha-synuclein accumulation in Parkinson′s disease (PD) as due to oxidative stress protein misfolding and alpha-synuclein aggregation in the dopaminergic neurons occurs (3, 6). The autophagy is a primary cellular catabolic program that is activated in response to oxidative stress and organelle lesion in the cell (7). p62 is bonded to the ubiquitinated proteins, and then it can be linked to LC3II and direct the ubiquitinated complex to be degraded in the autophagosomes. Meanwhile, after inducing oxidative stress the master transcription factor regulating cellular antioxidant defense system, known as Nrf2, is translocated to the nucleus and activates transcription of the genes containing the antioxidant response element (ARE) in their sequence (8). In this experimental study, the effects of trehalose on the survival and death of substantia nigra resident dopaminergic neurons, and also the cellular response to oxidative stress induced by 6-OHDA have been investigated in the course of the p62/Nrf2/ARE pathway which interconnects autophagy process with antioxidant response pathway in the mouse model of PD.

## Experimental

*Ethics statement*

The study was conducted in accordance with the guidelines for working with experimental animals set by the ethics committee (ethics code: IR.QUMS.REC.1396.148) of Qazvin University of Medical Sciences in accordance with the National Institute of Health Guide for the Care and Use of Laboratory Animals (NIH Publications No. 80-23) revised 1996.

*Animals*

Thirty male Wistar rats, weighing 180-250 g, were randomly selected and assigned into three experimental groups of a) control, b) lesion, and c) treatment (n = 10 for each group). All ethical guidelines were followed in order to reduce animal suffering. During the examinations, they maintained under a 12‌‌ h/12 h of light/dark cycle and given food and water *ad libitum*. 

*Parkinson’s disease model development*

For developing the Parkinson’s disease animal model, the rats were anesthetized by intraperitoneal injection of a mixture of Ketamine (100 mg kg^-1^) and Xylazine (5 mg kg^-1^). Their heads were then fixed in a stereotaxic device in accordance with the coordinates. The coordinates were set to 3 mm lateral to the left to cause a lesion, 4.5 mm abdominal from dura mater and +9.2 anterior-posterior to the interaural line. Incisor bar was also located 3.3 mm below the horizontal. After fixing the animal’s head on the device, the skin can be exposed by removing the hair of the head using regular razors and scissors. After finding the coordinates, the bone for injection was drilled at low speed in order to protect brain tissue from injury. In the control group, stereotaxic surgery was performed on the rats and 5 μL of a saline containing 0.2% of ascorbate was injected into the left corpus striatum by Hamilton syringe (10 μL). In the lesion group, 5 μL saline ascorbate 0.2% containing 25 μg of 6-OHDA was injected into the left corpus striatum (8, 9). In the treatment group, the rats were pretreated with trehalose 3% (Sigma-Aldrich) in drinking water for three days prior to the destruction of the corpus striatum (10). 

*Behavioral tests*

The behavioral test was performed on the rats in the three experimental groups before surgery and after four weeks afterward. Behavioral tests were implemented by intraperitoneal injection of apomorphine hydrochloride (Sigma-Aldrich) with the dose of 2.5 mg kg^-1^. Ten minutes before the surgery (baseline), the rats were kept in a cylindrical transparent chamber made of glass with the diameter of 33 cm and the height of 35 cm. After injecting medication, the total 360-degree rotation was measured manually for 60 min at the intervals of 10 min. The number of contralateral (opposite the lesion site or to the right) and the number of ipsilateral rotations (toward the lesion site or to the left side) were considered as positive and negative numbers, respectively. The net number of the rotations was calculated after subtracting rotations in two directions. 

*Nissl staining of the substantia nigra*

The rats were anesthetized by intraperitoneal injection of a mixture of Ketamine (100 mg kg^-1^) and Xylazine (5 mg kg^-1^) at week fourth, *i.e.* after performing the behavioral tests. The rats were perfused using normal saline and formalin. After perfusion, the brain was removed from the skull. For neuronal counts, tissue blocks were prepared from with the diameter of 10 μm from the substantia nigra of midbrain at intervals of 2.4 to 2.9 mm from the interaural point in accordance with the Paxinos atlas. The tissue sections were Nissl-stained with Cresyl violet solution (0.1%). The neurons in the dense part of the substantia nigra were counted in sections aligned with 4 levels of Paxinos atlas (*i.e.*, 2.96, 3.2, 3.8, and 4.2) as compared with the center of interaural line with the magnification of (×200, ×100). At each level, at least two sections were counted and the neurons with the cytoplasmic domain were also counted. Neurons in the dense part of the substantia nigra were counted in the sections aligned with 4 levels of Paxinos atlas (*i.e.*, 2.96, 3.2, 3.8, and 4.2) as compared to the center of the interaural line (Optical Microscope, × 200). At each level, at least two sections were counted.

*Assessment of DA and DOPAC levels*

After the end of the behavioral test, the brain was dissected from different groups in order to determine the DA and DOPAC (DA metabolite) levels. The substantia nigra was separated and homogenized by perchloric acid (0.17 M) at 10% w/v for 30 sec to extract DA and DOPAC from the tissues. Subsequently, the incubation was performed for 15 min, and the supernatant was poured into the microtube and centrifuged at 15,000 rpm for 15 min at 4 °C. The supernatant was then stored at -70 °C for subsequent analysis. The concentration of DA and DOPAC was measured by the high-performance liquid chromatography (HPLC) system (Waters Corporation, USA). Homogenates of nigral tissues (10%) were prepared in 0.1 M HClO4. After centrifugation at 4 °C for 15 min at 15,000 rpm, the supernatant was filtered (0.2 μm, Millipore), and a 20 μL sample was injected into a C18 column. The mobile phase was 0.163 M citric acid (pH 3.0), containing 0.02 mM NaCl with 0.69 mM sodium octanesulfonic acid (SOS) as the ion pairing reagent, 4% v/v acetonitrile and 1.7% v/v tetrahydrofuran DA and DOPAC were electrochemically detected, using an amperometric detector (Shimadzu, Japan). The level of monoamines was determined by comparison with freshly prepared standards, and their concentrations were expressed as ng/mg of tissue.

**Figure 1:**
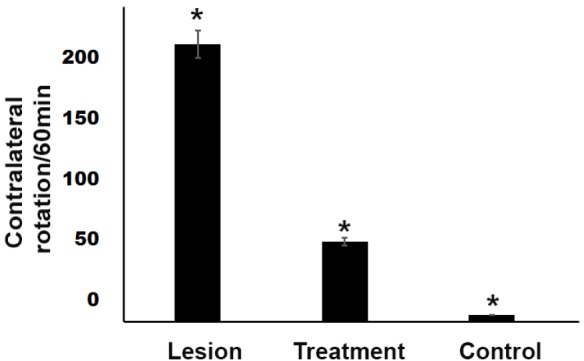
Amphetamine-induced contralateral rotations in rats lesioned with 6-OHDA. A significant protective effect (*P *< 0.05) of trehalose in the treatment group was observed in the apomorphine-induced rotational behavior. In the 6-OHDA- treated group, there was a significant increase (*P *< 0.05) in the number of rotations observed as compared with the control group. *P < 0.05

**Figure 2:**
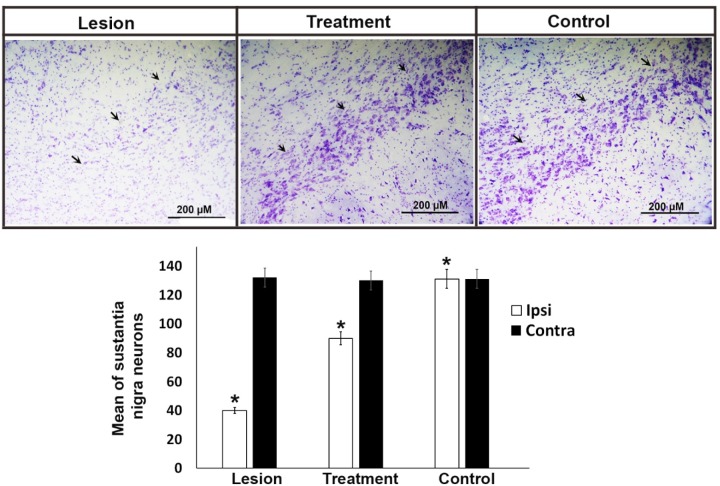
Representative Nissl staining of neurons and stereological quantification in the SNpc of the experimental groups. Arrows show the left substantia nigra in all experimental groups. In the lesion group, the neurons were significantly lower than other groups. The bar graph shows the average number of total cells obtained by stereological quantification of Nissl positive cells both in the contralateral and ipsilateral substantia nigra after 4 weeks of treatment. Injection of 6-OHDA in the striatum induced a significant decrease of neuronal populations in the substantia nigra regardless of the trehalose treatments. Values are mean ± SEM (n = 10), *P < 0.05, Scale bar, 200 μm

**Figure 3:**
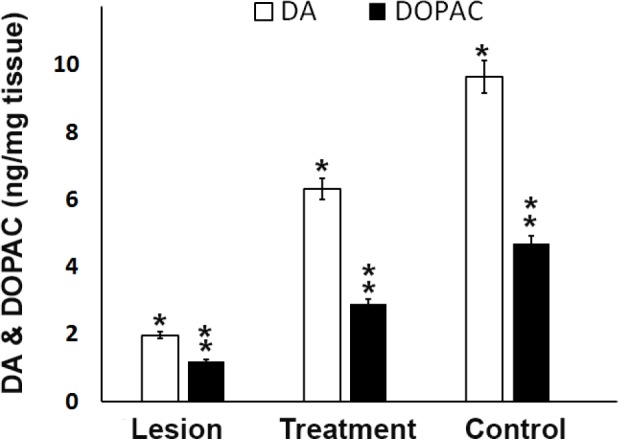
Dopamine (DA) and 3,4-dihydroxyphenylacetic acid (DOPAC) levels in the ipsilateral of striatum and SN from different groups. Analysis of substantia nigra DA and DOPAC concentrations showed trehalose treatment protects cells from 6-OHDA-induced of striatal dopamine and dopamine metabolites depletion. A significant decrease in rats that were exposed to 6-OHDA as compared to control and treatment rats was seen, *P < 0.05, ** *P *< 0.01

**Figure 4:**
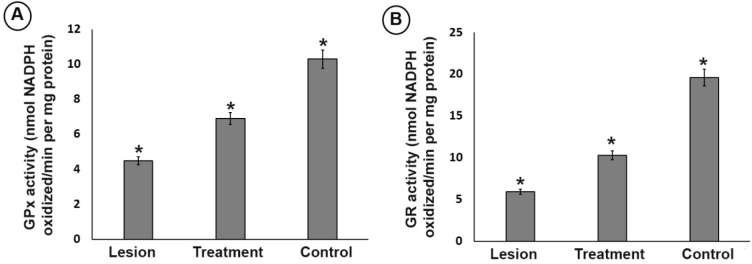
Effect of trehalose on the (A) glutathione peroxidase (GPx) and (B) glutathione reductase (GR) antioxidant enzyme activity in ipsilateral of striatum and substantia nigra from different groups. In the substantia nigra, the activity level of GPx and GR in the lesion group was significantly lower than that of the treatment and control groups. However, 3% trehalose in the treatment group inhibited further decrease in the GPx and GR than the lesion group. (*P < 0.05)

**Figure 5:**
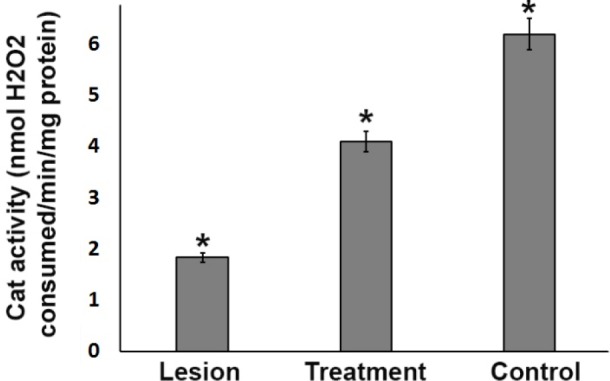
Catalase antioxidant enzyme activity in the ipsilateral substantia nigra (SN) from three experimental groups. In the SN, the CAT activity level in the lesion group was significantly lower than that in the treatment and control groups. However, 3% trehalose in the treatment group inhibited further reduction in CAT as compared to the lesion group, (*P < 0.05)

**Figure 6:**
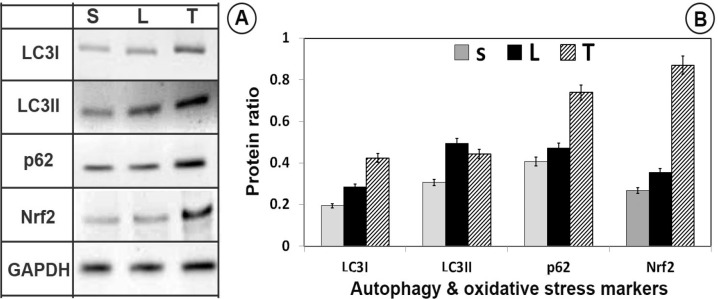
Western blot of autophagy (LC3I, LC3II and P62) and master antioxidant regulator (C-Nrf2, N-Nrf2) proteins. Trehalose activated autophagy and antioxidant defense system in SNpc. LC3/P62 immunoblotting was used to track the conversion of LC3I into LC3II and the expression of P62 for autophagic activity. (A) The immunoblotting of LC3-II/LC3-I, P62, C-Nrf2, N-Nrf2 and GAPDH from sham (S), lesion (L) and treatment (T) groups; (B) Quantitative analysis of immunoblotting of LC3-II/LC3-I, P62, C-Nrf2, N-Nrf2 controlled by GAPDH, respectively

*Assessment of glutathione peroxidase (GPx) activity*

GPx activity was estimated according to the previous studies (8, 11). To assess the GPx activity level, a mixture containing 100 μL of 1 M Tris-HCl (pH 8.0), 5 mM of EDTA, 20 μL of 0.1M GSH reductase (10 units/mL), 100 M of 2 mM NADPH, and 650 μL of distilled water, 10 μL of 7 mM tert-butyl hydroperoxide as well as 10 μL of supernatant containing the midbrain were used. The NADPH oxidation was measured at a wavelength of 340 nm using the spectrophotometric methods. A unit of activity was calculated as the amount of the GPx required to oxidize 1 μM of NADPH per minute. The enzyme activity was expressed as nmol of NADPH oxidized/min/mg of protein.

*Assessment of glutathione reductase (GR) activity*

The GR activity was assessed in accordance with the modified method by Mohandas and co-workers (11). The test was done via a 2 mL-mixture containing 1.65 mL of phosphate buffer (0.1 M, pH 7.6), 0.1 mL of NADPH (0.1 mM), 0.1 mL of EDTA (0.5 mM), 0.05 mL of oxidized glutathione (1 mM), and 0.1 mL of PMS (10% w/v). The level of NADPH oxidation at a wavelength of 340 nm was calculated as nmol of NADPH oxidized/min/mg of protein.

*Western blot*

The anti-LC3 (ab192890), anti-GAPDH (ab181602), anti-SQSTM1/p62 (ab56416) and anti Nrf2 (ab137550) primary antibodies (all provided from Abcam Company) were used for detecting the protein in the blots as previously reported with minor modifications (12). In brief, the protein extractions performed on ice using the lysis buffer from the experimental groups and protease inhibitor cocktail (Fermentas) were added immediately to the samples and protein concentrations assessed using Bradford assay. Equivalent amounts of each lysate were diluted in the sample buffer, boiled at 95 °C for 10 min and then subjected to SDS-PAGE using two separate gels with the gradient (running from 4 to 12% gel). The gels were transferred onto the nitrocellulose membrane (Millipore) by semi-dry transferring method (BioRad) and then, the membrane (Millipore) was blocked for 1 h at 37 °C in the Tris-Buffered Saline-Tween 20 (TBST) containing 5% blocking solution (Amersham). The membrane incubated for 1 h at room temperature in the TBST containing 5% blocking solution and the recommended dilution of the primary monoclonal antibody and then washed three times, 15 min each using TBST. It was then incubated for 1 h in the TBST containing 5% blocking solution containing 1:10,000 dilution of horseradish peroxidase-conjugated secondary antibody and after washing for three times, 15 min each with the TBST buffer, the membrane was then immersed in a mixture of equal volumes of ECL (Enhanced Chemiluminescence) detection solutions A and B (Amersham) and exposed to X-ray film in the dark room. The exposed films were developed using the specific solutions and then digitally photographed.

*Statistical analysis*

Each value is reported as the mean ± standard deviation. Significant differences were determined by one-way analysis of variance followed by Tukey’s multiple comparisons test. Differences with *P* < 0.05 were considered statistically significant.

## Results

*Apomorphine-induced behavioral testing*

The behavioral test was fulfilled in the first and fourth weeks after surgery. The results showed that all groups had a significant difference (*P *< 0.05) in the rotation number. The mean rotation number (Mean ± SEM) was 5, 56, and 193 four weeks after surgery in the control, treatment, and lesion groups, respectively. After operating and inducing the Parkinson′s disease model in the lesion group, the mean rotation number reached 193 in the animals, indicating the correct lesion in the animal model. In the treatment group, 3% trehalose reduced the damage to SN dopaminergic neurons, which was characterized by improved motor and reduced rotations in the treatment group compared to the lesion group. There was a significant difference (*P *< 0.05) in the rotation number between the treatment and the lesion groups (Figure 1).

*Histological examination by Nissl staining*

Following the behavioral tests, the animals were anesthetized to remove their midbrain. After preparation of the tissue block, neuron counting was performed by Nissl staining. The results indicated that the mean ± SEM neuronal counts in the right side (intact area) of substantia nigra was 131, 130, and 132 in the control, treatment, and lesion groups, with no significant difference between the groups, respectively. The mean ± SEM neuronal counts in the left side (degraded area) of the substantia nigra was 131, 90, and 42 in the control, treatment, and lesion groups, which was significantly different (*P* ≤ 0.05) among the groups (Figure 2). In the treatment group, 3% trehalose prevented the substantia nigra neuron apoptosis worsening and thus, the cells were less degenerated in this group in comparison with the lesion group. In the lesion group, the neuronal counts were significantly lower than that of the control group, indicating the lesion of SN neurons due to 6-OHDA usage.

*Assessment of DA and DOPAC levels*

The DA and DOPAC levels in the striatum and substantia nigra of the ipsilateral region in the three experimental groups were measured to evaluate the function and improvement of the dopaminergic neurons and the correlation between monoamine neurotransmitter alterations. As shown in Figure 3, the DA and DOPAC levels in the lesion group were significantly (*P* < 0.05) lower than those in the control group. However, the trehalose in the treatment group inhibited further reduction in DA and DOPAC levels as compared to the lesion group (Figure 3). Therefore, increasing DA and DOPAC levels in the treatment group in comparison with the lesion group indicate prolonged survival and improved function in the dopaminergic neurons of the substantia nigra.

*Assessment of GPx and GR activity level*

In the substantia nigra, the activity level of GPx and GR in the lesion group was significantly lower than that of the treatment and control groups (*P* < 0.05). However, 3% trehalose in the treatment group inhibited further decrease in the GPx and GR than the lesion group (Figure 4), highlighting the effect of trehalose on attenuating of oxidative stress.

*Assessment of catalase (CAT) activity level*

In the substantia nigra, the CAT activity level in the lesion group was significantly lower than that in the treatment and control groups (*P* < 0.05). However, 3% trehalose in the treatment group inhibited further reduction in CAT as compared to the lesion group (Figure 5), underlining the effects of trehalose on the oxidative stress reduction.

*Neuroprotective effects of trehalose by Nrf2 activation*

A rather significant increase in the GPx, CAT, and GR levels in the trehalose treatment groups were seen (Figures 4 and 5), thus it may possible that trehalose pretreatment neuroprotective actions may be mediated through Nrf2 master transcription factor translocation to the nucleus. As shown in Figure 6, trehalose induced nuclear translocation and accumulation of Nrf2 transcription factor. Thus, trehalose displays neuroprotective and antioxidant effects that appear to be related to Nrf2 activation.

*Neuroprotective effects of trehalose by autophagy induction*

To investigate the changes in autophagy, following trehalose treatment, LC3I, LC3II, and p62 protein levels were evaluated by western blot analysis. Compared with the sham-operated and 6-OHDA-lesion groups, LC3II/LC3I and p62 protein levels increased in the trehalose treatment group (*P *< 0.05, Figure 6).

## Discussion

According to the results in the present study, trehalose caused a) improved motor behavior and reduced apomorphine-induced rotations, b) reduced dopaminergic cell apoptosis of substantia nigra neurons caused by the 6-OHDA neurotoxin and, consequently, increased DA and DOPAC c) induced autophagy (increased LC3II/LC3I ratio), d) increased expression of p62 protein, e) elevated the Nrf2 transcription factor and its degredation inhibition and thus translocation into the cell nucleus f) increased expression of downstream antioxidant transcription factors, such as GR, GPx, and CAT and thus decreased ROS level. The behavioral and pathological tissue changes suggested the successful induction of the PD model. This unilateral PD induction is the most common pre-clinical model and the effects of this model on the dopaminergic system of the nigrostriatal pathway are well known (13-25). The 6-OHDA model renders molecular variations in the substantia nigra, which has the most similarity to the human PD (26). It is believed that 6-OHDA oxidized in the extracellular space produces the oxidative factors in the cell, induces oxidative stress in the cell, and eventually leads to mitochondrial electron transport chain dysfunction (24). As the 6-OHDA was injected to the left side of the brain, the asymmetrical damage to the dopaminergic neurons of substantia nigra was evaluated by the apomorphine-induced behavioral test (27). Apomorphine is a dopamine agonist and causes the contralateral rotation of the mouse towards the opposite side of the affected area as we assessed in the current study (24). In this study, the trehalose improved the motor function of the animals by reducing motor asymmetry and reducing rotations (28). The trehalose also prevented the reduction of DA and DOPAC levels, which could justify a decrease in motor symmetry in the treatment group in comparison with the lesion group. This finding is in agreement with the results of other researchers that DA and DOPAC contents are reduced by 6-OHDA neurotoxin. On the other hand, one of the important pathogenic factors in inducing the PD production is the oxidative stress. It occurs as the imbalance between the production and detoxification of free radicals in the cytoplasm is perturbed. The elevation in free radical levels leads to dysfunction and death of post-mitotic cells, such as neurons. Under the normal cellular circumstances, intracellular antioxidant defense system causes the removal of oxidative agents (29). The GPx and CAT detoxify dopaminergic cells from overproduced H_2_O_2_. Previous studies have demonstrated that the damage caused by 6-OHDA reduces the levels of antioxidant enzymes (30). In line with other studies, the ROS level was increased and GR, CAT and also GPx, DA and DOPAC levels were reduced in the lesion group, suggesting 6-OHDA-induced impairment in the antioxidant system. 6-OHDA also results in a consistent, significant decrease in the total GPx (Figure 4a) and GR activity (Figure 4b). Administration of trehalose can potently inhibit all of these pro-oxidative stress effects. These results imply that trehalose may be able to prevent oxidative damage in the SN after 6-OHDA exposure. Nrf2 activates expression of GPx and SOD enzymes which detoxifies ROS productions. We observed that trehalose increased the expression of Nrf2 that shows its regulatory effect at the translational level as well. Nrf2 activation neutralizes the neurotoxic agents in dopaminergic neurons such as the one that is related to 6-OHDA (31). We showed that the nigral Nrf2 level decreased in the lesion group and its levels increased with trehalose pretreatments. Considering the negative effects of 6-OHDA on Nrf2 expression, it is possible that trehalose prevents the 6-OHDA-induced oxidative stress by activating Nrf2. In the current study, trehalose treatment through antioxidant system enhancement prevented these reductions in levels or activities in the dopaminergic neurons and consequently improved the motor functions as seen in the apomorphine-behavioral tests (31). It has been well accepted that, Nrf2 is a master transcription factor responsible for gene expression of a series of anti-oxidant proteins and detoxifying enzymes in two ways known as canonical and non-canonical pathways. In the canonical pathway, Keap1, an adaptor protein of Cullin-3 ubiquitin ligase, senses electrophilic or oxidative stresses and then arrests ubiquitination of Nrf2 and inhibiting its degradation by the proteasome, leading to Nrf2 translocation and targeted gene expressions containing ARE in their sequence. Importantly, in addition to this canonical pathway, in a non-canonical mechanism of action, p62 competitively with Nrf2 to bind to Keap1 and p62 attachment to Keap1 releases Nrf2 from the Keap1 and hence, it translocates to the dopaminergic cell nucleus. Interestingly, this non-canonical pathway is hyper-activated in autophagy-deficient cells and tissues highlighting the p62-dependent of non-canonical antioxidant defense pathway (32, 33). Thus, the p62/SQSTM1-Keap1-Nrf2 axis may be linked to trehalose actions on increasing the cell survival as trehalose treatments increase p62 levels in the substantia nigra cells. Moreover, our results show that trehalose activates autophagy as well, as it increased the LC3II/LC3I ratio, indicating the activation of autophagy by the trehalose. In the autophagy, p62 acts as a link between LC3 and ubiquitinated substrates, which is degraded in autolysosome by autophagy, thus, autophagy activation decreases p62 levels in the dopaminergic cells and activating the autophagy and non-canonical Nrf2 pathway is simultaneously possible only under high levels of p62 gene expressions. Interestingly, the protein quantification results confirm that trehalose treatments increased p62 levels in the dopaminergic cells as high as to be effective in both of the processes, *i.e.* autophagy and non-canonical Nrf2 pathways which renders them be both active simultaneously in the cells. Recently, stem cell therapy approach, small molecules as drugs and epigenetic aspects are considered to find an effective way for the treatment of neurodegenerative disease (13, 34-43); however, investigations are continued to find more neurodegenerative-specific drugs.

## Conclusion

In the current study, trehalose simultaneously protects dopaminergic cells of the substantia nigra by activating both non-canonical p62/SQSTM1-Keap1-Nrf2 and autophagy pathways.
